# Supplementary material for: Genetic Variability, Character Association, and Path Coefficient Analysis in Transplant Aman Rice Genotypes
Source: Plants (Basel). 2022 Nov 2;11(21):2952. doi: 10.3390/plants11212952 (PMC9655179; doi:10.3390/plants11212952)
Supplement: Supplementary file 1 [file plants-11-02952-s001.zip › plants-1934614-supplementary.pdf]

Table S1. List of rice genotypes with their ancestry, biological status, and source of collection.

Entry	Genotypes	Cross combination	Generation	Source of Collection
1	Genotype-1	Rajasail × BRRI Dhan39	F_8	GPB, BSMRAU
2	Genotype-2	Pokkali × BRRI Dhan33	F_8	GPB, BSMRAU
3	Genotype-3	BR10 × BRRI Dhan33	F_8	GPB, BSMRAU
4	Genotype-4	BR10 × BRRI Dhan32	F_8	GPB, BSMRAU
5	Genotype-5	Pokkali × BR11	F_8	GPB, BSMRAU
6	Genotype-6	Pokkali × BRRI Dhan44	F_8	GPB, BSMRAU
7	Genotype-7	Rajasail × Pokkali	F_8	GPB, BSMRAU
8	Genotype-8	BR10 × Rajasail	F_8	GPB, BSMRAU
9	Genotype-9	Rajasail × BRRI Dhan44	F_8	GPB, BSMRAU
10	Genotype-10	BR11 × BRRI Dhan44	F_8	GPB, BSMRAU
11	Genotype-11	BRRI Dhan39 × BRRI Dhan32	F_8	GPB, BSMRAU
12	Genotype-12	BRRI Dhan33 × BRRI Dhan39	F_8	GPB, BSMRAU
13	Genotype-13	Rajasail × BRRI Dhan33	F_8	GPB, BSMRAU
14	Genotype-14	BRRI Dhan39 × BR10	F_8	GPB, BSMRAU
15	Genotype-15	Pokkali × BR10	F_8	GPB, BSMRAU
16	Genotype-16	BRRI Dhan39 × Pokkali	F_8	GPB, BSMRAU
17	Genotype-17	BRRI Dhan33 × BRRI Dhan44	F_8	GPB, BSMRAU
18	Genotype-18	BRRI Dhan39 × BR11	F_8	GPB, BSMRAU
19	Genotype-19	BR11 × BRRI Dhan33	F_8	GPB, BSMRAU
20	Genotype-20	BRRI Dhan33 × BRRI Dhan32	F_8	GPB, BSMRAU

GPB; Genetics and plant breeding department, BSMRAU; Bangabandhu Sheikh Mujibur Rahman Agricultural University.

Table S2. Principal component (PC) coefficients of all traits.

For each variable, the highest loading values for the first three components are in bold.

Traits/Components	PC1	PC2	PC3
DFF	-0.22436	0.148873	**-0.3534**
PH	**0.640718**	0.614163	0.19061
NET	**0.680142**	-0.21804	-0.38012
FLL	**0.607936**	0.514807	0.357468
PL	**0.738328**	0.210246	0.422209
NPB	-0.06648	-0.33069	**0.767217**
NSB	**0.427081**	-0.3356	0.264764
NFS	**0.738274**	-0.61489	0.053047
SS	**-0.65577**	0.261572	0.205934
TGW	-0.17674	**0.891421**	-0.03586
HI	**0.816269**	0.137696	-0.19099
GY	**0.892616**	0.125451	-0.32326
Proportion of variance (%)	37.45303	18.78294	12.14971
Cumulative proportion (%)	37.45303	56.23597	68.38568

DFF-days to 50% flowering; PH-plant height; NET-number of effective tillers per hill; FLL-flag leaf length; PL-panicle length; PBP- number of primary branches per panicle; SBP-number of secondary branches per panicle; NFS-number of filled spikelets per panicle, SS-spikelet sterility, TGW-1000-grain weight; HI-harvest index.
